# Selection of Suitable Reference Genes for Quantitative Real-Time PCR Normalization in Three Types of Rat Adipose Tissue

**DOI:** 10.3390/ijms17060968

**Published:** 2016-06-22

**Authors:** Wan-Xia Zhang, Jie Fan, Jing Ma, Yi-Song Rao, Li Zhang, You-E Yan

**Affiliations:** 1 Department of Pharmacology, Basic Medical School of Wuhan University, 185, DongHu Road, Wuhan 430071, China; zhangwanxia@whu.edu.cn (W.-X.Z.); fanjcn@163.com (J.F.); raoyisong@whu.edu.cn (Y.-S.R.); alicedoupont@163.com (L.Z.); 2 Department of Anesthesiology, Zhongnan Hospital of Wuhan University, Wuhan 430071, China; mj0817@gmail.com

**Keywords:** reference gene, geNorm, NormFinder, BestKeeper, rat adipose tissue

## Abstract

Quantitative real-time PCR (qRT-PCR) is the most classical technique in the field of gene expression study. This method requires an appropriate reference gene to normalize mRNA levels. In this study, the expression stability of four frequently-used reference genes in epididymal white adipose tissue (eWAT), inguinal beige adipose tissue (iBeAT) and brown adipose tissue (BAT) from obese and lean rats were evaluated by geNorm, NormFinder and BestKeeper. Based on the Minimum Information for Publication of Quantitative Real-Time PCR Experiments (MIQE) guidelines, the two most stable reference genes were recommended in each type of adipose tissue. Two target genes were applied to test the stability of the reference genes. The geNorm and NormFinder results revealed that *GAPDH* and *36B4* exhibited the highest expression stabilities in eWAT, while *36B4* and *β-actin* had the highest expression stabilities in iBeAT and BAT. According to the results of the BestKeeper analysis, *36B4* was the most stable gene in eWAT, iBeAT and BAT, in terms of the coefficient of variance. In terms of the coefficient of correlation, *GAPDH*, *36B4* and *β-actin* were the most stable genes in eWAT, iBeAT and BAT, respectively. Additionally, expected results and statistical significance were obtained using a combination of two suitable reference genes for data normalization. In conclusion, *36B4* and *GAPDH*, in combination, are the best reference genes for eWAT, while *36B4* and *β-actin* are two most suitable reference genes for both iBeAT and BAT. We recommend using these reference genes accordingly.

## 1. Introduction

The gradually increasing prevalence of obesity has caused adipose tissue to be a focus. Adipose tissue, which has been divided into two main types, white adipose tissue (WAT) and brown adipose tissue (BAT), is a complex and highly active metabolic organ [1]. WAT, best known for its role in the storage of energy, is distributed mainly in visceral and subcutaneous sites. BAT is viewed as a key site of nonshivering thermogenesis and has been considered an attractive target for treating obesity and obesity-associated diseases [2]. Most BAT depots are located in the interscapular and perirenal regions in rodents and large mammals [3]. Recently, a third class of adipocytes was discovered in WAT, especially in inguinal WAT, and the cells were termed “beige” or “brown-like” adipocytes [3]. Interestingly, even though beige adipocytes exist in WAT, beige and brown adipocytes have similar morphology, in the form of multilocular lipid droplets, and a common ability to carry out thermogenesis [2]. In fact, increasing evidence suggested that WAT browning and BAT activation may be promising strategies for treating obesity [4,5,6]. However, there is still relatively little knowledge of the relations between WAT and BAT, especially at the molecular level. In order to study depot-related differences in mRNA expression in adipose tissue, a sensitive and specific technique, such as quantitative real-time polymerase chain reaction (qRT-PCR) is required [7].

Actually, qRT-PCR is a valuable tool for gene expression analysis and is used in many clinical and scientific research fields [8]. However, many studies have demonstrated that the results of qRT-PCR are affected by experimental operation, original sample amount, RNA integrity, cDNA quality and the efficiency of reverse transcription [9,10]. Thus, choosing a stable reference gene for data normalization is the most primary step of a qRT-PCR assay [11]. However, no universal reference gene has been confirmed, and reference genes are regulated by the certain experimental conditions [12]. Therefore, the stability of frequently-used reference genes is relative. From a survey of different studies of adipose tissue, qRT-PCR data were normalized to different reference genes, such as Glyceraldehyde-3-phosphate dehydrogenase (*GAPDH*) [13], *β-actin* [14], *18S* [15] and acidic ribosomal phosphoprotein P0 (*36B4*) [16]. However, no research has studied the stability of frequently-used reference genes in different types of rat adipose tissue.

Since 2002, several mathematical algorithms, such as geNorm [17], NormFinder [10], and BestKeeper [18], have been applied to evaluate the expression stability of reference genes. GeNorm evaluates the most stable reference genes for normalization determined by the M value, which is calculated from the arithmetic mean of pairwise variations of each gene. NormFinder validates the expression stability of each reference gene and consider both intra- and intergroup variations for normalization. BestKeeper calculates the expression variability of reference genes based on the standard deviation (SD), coefficient of variance (CV) and coefficient of correlation. In the comprehensive ranking, the top ranked genes are recommended as reference genes for data normalization in similar experimental systems.

In 2009, the Minimum Information for Publication of Quantitative Real-Time PCR Experiments (MIQE) guidelines proposed that selecting multiple suitable reference genes for reliable normalization in qRT-PCR is necessary [19]. Therefore, in this study, we used geNorm, NormFinder and BestKeeper to identify four commonly used reference genes in epididymal WAT (eWAT), inguinal beige adipose tissues (iBeAT) and brown adipose tissue (BAT) from obese and lean rats, and to identify the two most suitable reference genes for data normalization. Furthermore, we used two target genes to confirm how the different reference genes affect the final results.

## 2. Results

### 2.1. Body Weight and Abdomen Circumference

**Figure 1:**
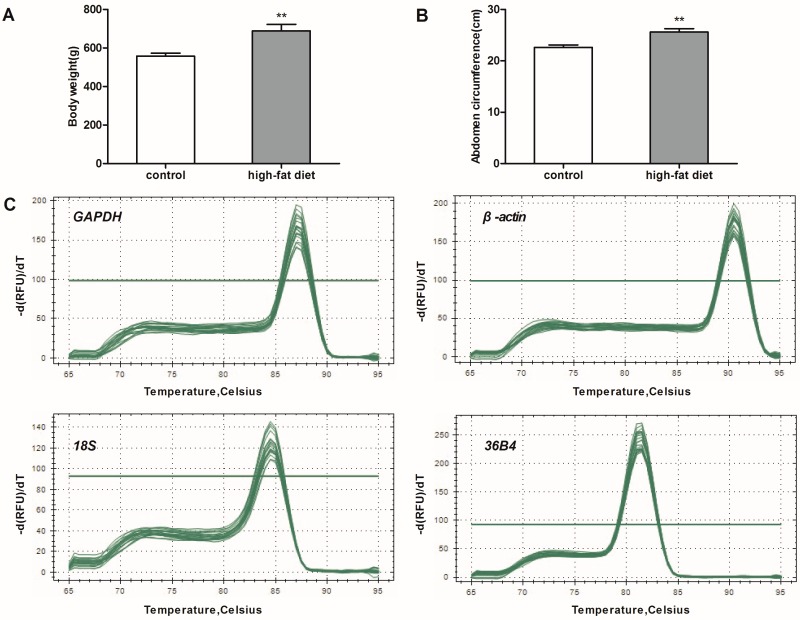
Body weight, abdominal circumference and melting curves for the quantitative real-time polymerase chain reaction (qRT-PCR) products. (**A**) body weight (*n* = 6); (**B**) abdomen circumference (*n* = 6); (**C**) melting curves for the qRT-PCR products. The single sharp peak represents a specific qRT-PCR product. *GAPDH*: Glyceraldehyde-3-phosphate dehydrogenase; *36B4*: acidic ribosomal phosphoprotein P0. ** *p* < 0.01 *vs.* control.

Compared to the control, rats with high-fat diets had a higher body weight (+23%, *p* < 0.01, Figure 1A) at 26 weeks old. Similarly, the abdomen circumference of rats with high-fat diets was significantly increased (+13%, *p* < 0.01, Figure 1B).

### 2.2. RNA Purity and Primer Specificity

The RNA purity, which was monitored by 260/280 absorbance (A_260_/A_280_) ratios, ranged from 1.86 to 2.01. Therefore, the purity of the isolated RNA from different types of adipose tissue (eWAT, iBeAT and BAT) in control and high-fat diet rats was satisfactory for further studies. The specificity of each primer pair was verified by melting curves analysis using a corresponding cDNA sample, only a single qRT-PCR product was amplified (Figure 1C).

### 2.3. Expression Levels of the Reference Genes

**Figure 2:**
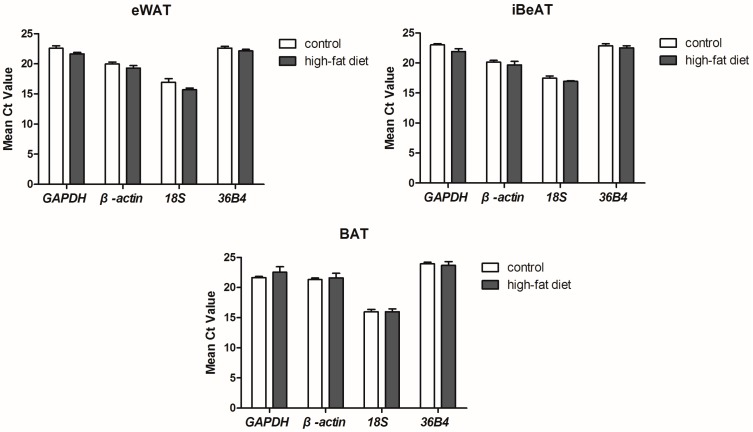
Expression levels of the reference genes in different types of adipose tissues between control and high-fat diet groups (mean ± SEM, *n* = 6). *GAPDH*: Glyceraldehyde-3-phosphate dehydrogenase; *36B4*: acidic ribosomal phosphoprotein P0; eWAT: epididymal white adipose tissue; iBeAT: inguinal beige adipose tissues; BAT: brown adipose tissue.

The cycle threshold (*C*_t_) value of each reference gene was obtained from qRT-PCR analysis (Figure 2). No significant differences between control and high-fat diet groups were found for all four reference genes in three different types of adipose tissue. The mean *C*_t_ value of the reference genes ranged from 16 to 23 cycles, with *18S* having the highest (16.42 ± 0.19) and *36B4* having the lowest (22.96 ± 0.18) transcript levels. The obtained *C*_t_ values were applied to evaluate the expression stability of the reference genes with the geNorm [17], NormFinder [10] and BestKeeper [18], respectively.

### 2.4. Expression Stability of the Reference Genes

#### 2.4.1. GeNorm Analysis

**Figure 3:**
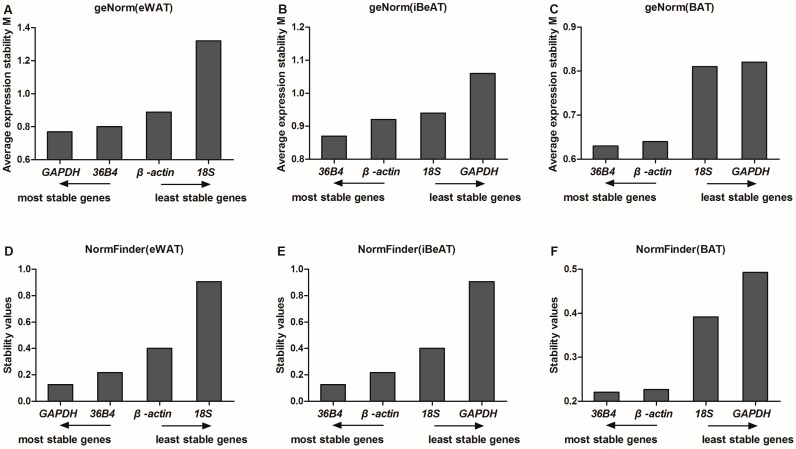
Expression stability values and ranking of the reference genes in different types of adipose tissue by geNorm and NormFinder (*n* = 12). (**A**–**C**) gene expression stability values (M) in epididymal white adipose tissue (eWAT), inguinal beige adipose tissues (iBeAT) and brown adipose tissue (BAT) by geNorm; (**D**–**F**) gene expression stability values in eWAT, iBeAT and BAT by NormFinder. *GAPDH*: Glyceraldehyde-3-phosphate dehydrogenase; *36B4*: acidic ribosomal phosphoprotein P0.

The geNorm analysis (Figure 3A–C) demonstrated that all of the reference genes had an M value below 1.5, which is the maximum value for acceptable gene expression stability as defined by Vandesompele *et al*. [17]. The gene with the lowest M value has the most stable expression. As shown in Figure 3A, the ranking of M value in eWAT was as follows: *GAPDH* > *36B4* > *β-actin*> *18S*. *GAPDH* (*M* = 0.77) and *36B4* (*M* = 0.80) were the two most stable reference genes, while *18S* was the least stable reference gene in eWAT. The results obtained from geNorm in iBeAT and BAT were the same. Ranking was listed as follows: 3*6B4* > *β-actin*> *18S*> *GAPDH*. *36B4* (*M* = 0.87; *M* = 0.63) and *β-actin* (*M* = 0.92; *M* = 0.64) were identified as the two most stable reference genes and *GAPDH* (*M* = 1.06; *M* = 0.82) was the least stable reference gene in iBeAT and BAT (Figure 3B,C).

#### 2.4.2. NormFinder Analysis

By using the NormFinder method, lower stability values correspond to lower variation, and hence, higher expression stability of gene [10]. In our study, NormFinder analysis showed that *GAPDH* and *36B4* had the lowest stability values (0.127 and 0.217, respectively) and were the two most stable reference genes in eWAT, while *18S* was ranked as the least stable gene (stability values of 0.906) (Figure 3D). However, in iBeAT and BAT, *36B4* and *β-actin* had the lowest stability values (0.348 and 0.448; 0.221 and 0.227, respectively) and were the most two stable reference genes, whereas *GAPDH* was the least stable gene (stability values of 0.608 and 0.493, respectively) (Figure 3E,F). The results of NormFinder analysis were highly consistent with the geNorm analysis.

#### 2.4.3. BestKeeper Analysis

According to the BestKeeper analysis, it was unacceptable that genes show an SD higher than one [18]. Thus, in our study, *18S* (SD = 1.05) in eWAT and *GAPDH* (SD = 1.12) in BAT were both eliminated (Table 1 and Table 2). Furthermore, *β-actin* was excluded for further analysis in iBeAT because it had the highest variation (CV = 4.45) (Table 3). The ranking of reference genes by CV has a subtle distinction from ranking by coefficient of correlation. Based on the results of CV calculation, the most stable gene to the least stable gene were ranked as follows: *36B4* > *GAPGH* > *β-actin* (eWAT) (Table 1); *36B4* > *β-actin* > *18S* (BAT) (Table 2); *36B4* > *GAPGH* > *18S* (iBeAT) (Table 3). However, when ordered by the coefficient of correlation, the results were as follows: *GAPGH* > *36B4* > *β-actin* (eWAT) (Table 1); *β-actin* > *36B4* > *18S* (BAT) (Table 2); *36B4* > *18S* > *GAPDH* (iBeAT) (Table 3). As a result, in this study, BestKeeper cannot provide a definite conclusion.

#### 2.4.4. Comprehensive Ranking of the Reference Genes

Different rankings of the most suitable reference genes were obtained from geNorm, NormFinder and BestKeeper. This difference in ranking has also been mentioned in other articles [20,21,22]. Based on three different methods, each reference gene was ranked from 1 (most stable) to 4 (least stable). The arithmetic mean value of each gene was obtained, and the comprehensive stability ranking of the reference genes were shown in Table 4, Table 5 and Table 6. *36B4* exhibited a higher stability of gene expression in each type of adipose tissue (eWAT, iBeAT and BAT). In accordance with the MIQE guidelines, multiple reference genes for normalization in qRT-PCR were then proposed*.* In our study, the most two suitable reference genes for eWAT are *GAPDH* and *36B4*, while *36B4* and *β-actin* are the most two suitable reference genes for both iBeAT and BAT (Table 4, Table 5 and Table 6).

### 2.5. Normalization of Two Target Genes with Reference Genes

**Figure 4:**
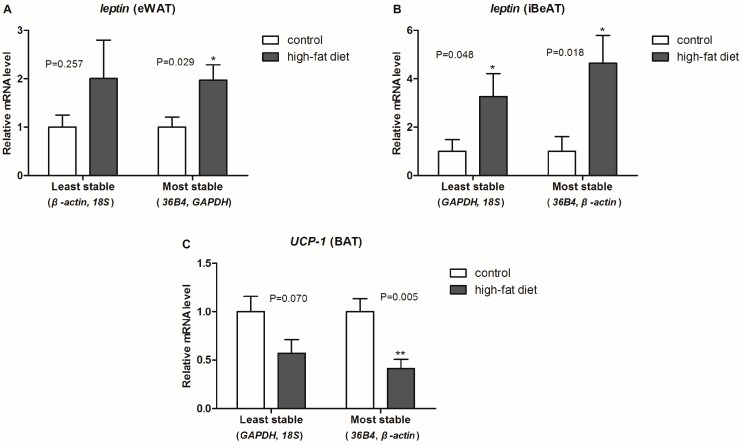
Relative mRNA levels of two target genes in different adipose tissue. (mean ± SEM, *n* = 6). The relative mRNA levels of two target genes, normalized with the geometric means of the abundance of the most or least stable reference genes, in different adipose tissue. (**A**) the relative mRNA levels of *leptin* in epididymal white adipose tissue (eWAT); (**B**) the relative mRNA levels of *leptin* in inguinal beige adipose tissues (iBeAT); (**C**) the relative mRNA levels of uncoupling protein 1 *(UCP-1)* in brown adipose tissue (BAT). *GAPDH*: Glyceraldehyde-3-phosphate dehydrogenase; *36B4*: acidic ribosomal phosphoprotein P0. * *p* < 0.05, ** *p* < 0.01 *vs.* control.

To identify how different reference genes affect the final results, the gene for the adipose-derived cytokine *leptin* was normalized with the combination of most or least stable reference genes. As shown in Figure 4A,B, the mRNA levels of *leptin* in eWAT, when calculated with the combination of most stable reference genes (*36B4* and *GAPDH*), were significantly increased (+97%, *p* = 0.029) in obese rats, while they were not significantly changed when calculated with the combination of least stable reference genes (*β-actin* and *18S*). When using the combination of the most stable reference genes (*36B4* and *β-actin*) for normalization, the mRNA levels of *leptin* in iBeAT were significantly higher (*p* = 0.018) in obese rats. However, when using the combination of least reference genes (*18S* and *GAPDH*) for normalization, the mRNA levels of *leptin* in iBeAT showed significantly less increases (*p* *=* 0.048) in obese rats.

Uncoupling protein 1 (*UCP-1*), a brown fat-specific gene, is an energy metabolism-related factor that plays a central regulator role in mitochondrial biogenesis and thermogenesis in BAT [23]. In our study, we observed significantly decreased (−59%, *p* = 0.005) mRNA levels of *UCP-1* in obese rats when calculated with the combination of most stable reference genes (*36B4* and *β-actin*). However, when calculated with the combination of least stable reference genes (*18S* and *GAPDH*), there were no significant changes (−43%, *p* *=* 0.070) in the *UCP-1* mRNA levels in obese rats (Figure 4C). Therefore, a stable reference gene improves the accuracy of a gene expression study.

## 3. Discussion

In molecular biological research, gene expression analysis is one of the most frequently-used strategies in the field of gene study involved in signaling and metabolic pathways. It is of vital significance to select an appropriate reference gene for the normalization of gene expression. It is generally known that *GAPDH* and *β-actin* are the two most commonly-used reference genes. However, some researchers have identified that *GAPDH* and *β-actin* showed a significant variation in different cells, tissues and experimental conditions [24,25,26]. Furthermore, Catalan and his colleagues showed that *GAPDH* is a less appropriate reference gene in human omental and subcutaneous adipose tissue from obesity and type 2 diabetes patients, because of the variational expression [27]. Therefore, we identified the expression stability of four reference genes (*GAPDH*, *β-actin*, *36B4* and *18S*) in three types of rat adipose tissue (eWAT, iBeAT and BAT) by using different software: geNorm, NormFinder and BestKeeper. In addition, in accordance with the MIQE guidelines, the two most suitable reference genes were recommended in each type of adipose tissue.

GeNorm software is one of the most commonly-used algorithms applied to evaluate reference gene expression stability via a pairwise comparison between one reference gene and each other reference gene from each sample [17]. In our study, *GAPDH* and *36B4* appeared to be the two most stable reference genes in eWAT among lean and obese rats. However, *GAPDH* was the least stable reference gene in both iBeAT and BAT. Therefore, *GAPDH* is not a universal reference gene in adipose tissue. This finding is consistent with a previous study by Akamine et al*.*, who verified that *GAPDH* was not suitable as a universal reference gene in adipose tissue because of its inconsistent expression levels between WAT and BAT [28]. In our study, however, we found that *36B4* and *β-actin* had highest expression stability in both iBeAT and BAT. It is suggested that *36B4* is the universal reference gene in different types of adipose tissue.

Unlike geNorm, the NormFinder procedure directly and robustly estimates gene expression stability and ranks reference genes depend on the variation within the intra- and the inter-group. As Andersen mentioned in his report, the NormFinder procedure focuses on differences between sample subgroups, and the result is less affected by the correlated expression of the reference genes [10]. In our study, the results obtained from NormFinder analysis were quite consistent with the geNorm analysis. *GAPDH* is the most stable reference gene, followed by *36B4* in eWAT. *36B4* and *β-actin* were the most stable reference genes in both iBeAT and BAT.

BestKeeper is another traditional software that is used to assess the stability of gene expression. In the BestKeeper procedure, evaluating the stability of gene expression was co-determined by three essential criteria, which are the SD, CV and the coefficient of correlation [18]. In our study, *36B4* was the most stable gene in eWAT based on the CV value, while *GAPDH* and *β-actin* were ranked as the second and third, respectively; these results were different from the geNorm and NormFinder analysis. In terms of the coefficient of correlation, however, *GAPDH* was the most stable gene in eWAT. In addition, this result completely supports the geNorm and NormFinder analysis. In iBeAT, *36B4* had a highest expression stability based on both CV value and coefficient of correlation. This result was consistent with the geNorm and NormFinder analysis. In BAT, a difference was also observed based these two criteria. When ordered by CV value and coefficient of correlation, *36B4* and *β-actin* were the most stable genes, respectively.

To evaluate the different results from the three algorithms systematically, a comprehensive ranking of each reference gene was calculated. Results show that *GAPDH* was the most stable reference gene in eWAT, but it had the highest variability in both iBeAT and BAT. In addition, *36B4* was a highly stable gene in each type of adipose tissue, even though the results from the geNorm and NormFinder analyses had a tiny disparity with *GAPDH* in eWAT. For a realistic study of obesity, more than one type of adipose tissue should be considered. Based on new research, “browning” of WAT will become a new strategy for obesity treatment [4,29]. Therefore, considering the variety of experimental subjects and practicability, a relatively universal reference gene among each type of adipose tissue should be used. According to our study, *36B4* is the universal reference gene in adipose tissue. In accordance with the MIQE guidelines, however, the use of multiple reference genes for normalization in qRT-PCR were proposed*.* We recommend *36B4* and *GAPDH* as the best combination of reference genes for eWAT, and *36B4* and *β-actin* as the most suitable reference genes for both iBeAT and BAT. To further validate our assumptions in an actual experiment, we used either the combination of most or the least stable reference gene to normalize same target genes (e.g., *leptin* and *UCP-1*). As shown in Figure 4, expected results and statistical significance were obtained by normalizing with the two most stable reference genes. This result totally confirmed our assumptions.

## 4. Experimental Section

### 4.1. Animals

Six-week-old male Wistar rats weighing 150–200 g were provied by the Experimental Center of Hubei Medical Scientific Academy (No. 2008-0005, Wuhan, Hubei, China). Rats were maintained with 12 h light-dark cycles at 22–24 °C room temperature. Rats were randomly assigned to two groups: a high-fat diet group (*n* = 6) and a control group (*n* = 6). The rats in the control group were fed a standard chow diet and water ad libitum. Meanwhile, rats in high-fat diet group were fed a high-fat diet (45% calories from fat, D12451) for 20 weeks. The body weight of each rat was monitored weekly until 26 weeks of age. At the end of the experiment, rats fasted for 12 h and were then placed in a separate quiet room for anesthesia. After the disappearance of the righting reflex, the abdominal circumference of animal was measured. Rats were killed by rapid decapitation. eWAT, iBeAT and BAT were collected. Adipose tissues were immediately frozen in liquid nitrogen and later stored at −80 °C for subsequent experiments.

### 4.2. RNA Isolation, Quantification and cDNA Synthesis

The total RNA of eWAT, iBeAT and BAT were extracted using the Trizol instructed by the manufacturer’s protocol. The oil was removed by repeated centrifugation. The purity of the total RNA was performed on a NanoDrop 2000c spectrophotometer (Thermo Scientific, Wilmington, DE, USA). Only RNA samples with a 260/280 ratio between 1.8 and 2.1 were used in subsequent analyses. cDNA was synthesized from 1 μg of total RNA from eWAT, iBeAT and BAT in control and high-fat diet rats using the PrimeScript RT-PCR kit (Takara Biotech, Kusatsu, Japan) instructed by the manufacturer’s recommendations. After synthesis, the template for qRT-PCR was prepared for further studies by diluting the obtained cDNA tenfold with ultrapure water.

### 4.3. qRT-PCR

qRT-PCR was conducted on a StepOnePlus™ real-time PCR system (Applied BioSystems, Foster City, CA, USA). Each reaction contained 1 μL of cDNA template and 9 μL of the reaction mixture, which were used in the SYBR Green Core Reagent kit (Applied Biosystems, Foster City, CA, USA). Relative mRNA expression levels were calculated by the ΔΔ*C*_t_ method. The cycling condition is as follows: pre-denaturation, 95 °C for 30 s; denaturation, 95 °C for 10 s; annealing, 60 °C for 30 s; 40 cycles. Then, a melting curve was generated by increasing the temperature from 65 to 95 °C with continuous collection of the SYBR Green fluorescence signal. All primer sequences and relevant parameters about the genes are showed in Table 7.

### 4.4. Reference Gene Expression Stability Analysis

The initial qRT-PCR data were obtained from the StepOnePlus™ software (Applied BioSystems, Foster City, CA, USA) and put into an Excel datasheet. Then, statistical tools such as GeNorm [17], NormFinder [10] and BestKeeper Version [18] were used to evaluate the stability of gene expression.

### 4.5. Statistical Analysis

Data were presented as the mean ± SEM. Graphics were performed using Prism (GraphPad Software, Version 5.0, La Jolla, CA, USA). All results were evaluated using an unpaired Student’s *t*-test, and differences with *p* < 0.05 were regarded as statistically significant.

## 5. Conclusions

Combining all of the various software tested, MIQE guidelines and experimental verification, *36B4* and *GAPDH* were recommended as the optimal combination of reference genes for eWAT, and *36B4* and *β-actin* were found to be the optimal combination of reference genes for both iBeAT and BAT.

## Figures and Tables

**Table 1:** Expression stability of four reference gene in epididymal white adipose tissue (eWAT) evaluated by BestKeeper (*n* = 12).

Gene	Geometric Mean (*C*_t_)	Arithmetic Mean (*C*_t_)	Minimum (*C*_t_)	Maximum (*C*_t_)	Standard Deviation (±*C*_t_)	Coefficient of Variance (% *C*_t_)	Correlation Coefficients (*r*)
*36B4*	22.38	22.39	21.24	23.69	0.53	2.36	0.789
*GAPDH*	22.10	22.11	20.76	23.91	0.69	3.12	0.878
*β-actin*	19.60	19.62	17.77	20.99	0.76	3.86	0.788
*18S*	16.27	16.31	14.65	18.82	1.05	6.44	0.705

**Table 2:** Expression stability of four reference gene in brown adipose tissue (BAT) evaluated by BestKeeper (*n* = 12).

Gene	Geometric Mean (*C*_t_)	Arithmetic Mean (*C*_t_)	Minimum (*C*_t_)	Maximum (*C*_t_)	Standard Deviation (±*C*_t_)	Coefficient of Variance (% *C*_t_)	Correlation Coefficients (*r*)
*36B4*	23.78	23.80	20.89	25.26	0.78	3.26	0.960
*β-actin*	21.42	21.46	18.48	23.94	0.94	4.38	0.965
*18S*	15.93	15.96	13.74	17.99	0.75	4.68	0.933
*GAPDH*	22.03	22.08	18.45	24.90	1.12	5.06	0.953

**Table 3:** Expression stability of four reference gene in inguinal beige adipose tissues (iBeAT) evaluated by BestKeeper (*n* = 12).

Gene	Geometric Mean (*C*_t_)	Arithmetic Mean (*C*_t_)	Minimum (*C*_t_)	Maximum (*C*_t_)	Standard Deviation (±*C*_t_)	Coefficient of Variance (% *C*_t_)	Correlation Coefficients (*r*)
*36B4*	22.66	22.68	20.93	23.67	0.67	2.97	0.798
*GAPDH*	22.43	22.45	19.92	23.75	0.73	3.23	0.712
*18S*	16.97	16.98	15.99	18.41	0.66	3.87	0.751
*β-actin*	19.88	19.91	17.89	21.93	0.89	4.45	0.864

**Table 4:** Comprehensive ranking (eWAT).

Ranking Order	geNorm	NormFinder	BestKeeper		Comprehensive Ranking (Mean Rank Value)
			CV	r	
1	*GAPDH*	*GAPDH*	*36B4*	*GAPDH*	*GAPDH* (*1.25*)
2	*36B4*	*36B4*	*GAPDH*	*36B4*	*36B4* (*1.75*)
3	*β-actin*	*β-actin*	*β-actin*	*β-actin*	*β-actin* (*3.00*)
4	*18S*	*18S*	*-*	*-*	*18S* (*4.00*)

**Table 5:** Comprehensive ranking (iBeAT).

Ranking Order	GeNorm	NormFinder	BestKeeper		Comprehensive Ranking (Mean Rank Value)
			CV	r	
1	*36B4*	*36B4*	*36B4*	*36B4*	*36B4* (*1.00)*
2	*β-actin*	*β-actin*	*GAPDH*	*18S*	*β-actin* (*2.00*)
3	*18S*	*18S*	*18S*	*GAPDH*	*18S* (*3.00*)
4	*GAPDH*	*GAPDH*	*-*	*-*	*GAPDH* (*4.00*)

**Table 6:** Comprehensive ranking (BAT).

Ranking Order	GeNorm	NormFinder	BestKeeper		Comprehensive Ranking (Mean Rank Value)
			CV	r	
1	*36B4*	*36B4*	*36B4*	*β-actin*	*36B4* (*1.25*)
2	*β-actin*	*β-actin*	*β-actin*	*36B4*	*β-actin* (*1.75*)
3	*18S*	*18S*	*18S*	*18S*	*18S* (*3.00*)
4	*GAPDH*	*GAPDH*	*-*	*-*	*GAPDH* (*4.00*)

**Table 7:** Descriptions of reference genes and parameters derived from quantitative real-time polymerase chain reaction analysis.

Genes	Gene Function	Gene Accession Number	Primer Sequence (Forward/Reverse)	Tm (°C)	Product (bp)	Amplification Efficiency (%)	*R*^2^
*GAPDH*	glycolysis, glucose metabolism	NM 017008.4	TGCCACTCAGAAGACTGTGG/TTCAGCTCTGGGATGACCTT	60 °C	129	99.1	0.994
*18S*	ribosomal protein	NR 046237.1	GGAGAGGGAGCCTGAGAAAC/CAATTACAGGGCCTCGAAAG	60 °C	128	109.7	0.994
*β-actin*	structural constituent of cytoskeleton	NM 031144.3	ATGGATGACGATATCGCTGC/CTTCTGACCCATACCCACCA	60 °C	150	93.4	0.994
*36B4*	structural constituent of ribosome	XM 015505154.1	CGACCTGGAAGTCCAACTAC/ATCTGCTGCATCTGCTTG	60 °C	109	100.2	0.997
*leptin*	regulates food intake and energy metabolism	NM 013076.3	TTTCACACACGCAGTCGGTATC/GGTCTGGTCCATCTTGGACAAA	60 °C	101	99.2	0.997
*UCP-1*	participate in nonshivering thermogenesis	NM 012682.2	GCCTCTACGATACGGTCCAA/TGCATTCTGACCTTCACCAC	60 °C	145	93.4	0.997
